# Technological evolution of *in vitro* mock circulatory loops for advanced cardiovascular assist device evaluation

**DOI:** 10.3389/fphys.2025.1610040

**Published:** 2025-09-02

**Authors:** Hongyu Li, Yiwen Wang, Xuefeng Wu, Lijie Zhou, Lijia Liu

**Affiliations:** School of Mechanical and Power Engineering, Harbin University of Science and Technology, Harbin, China

**Keywords:** mock circulatory loop, cardiovascular assist devices, hemodynamics, systemic circulation, pulmonary circulation, hybrid-MCL

## Abstract

The clinical reliability of implantable cardiovascular assist devices (CADs) necessitates rigorous verification by the Mock Circulatory Loop (MCL) to assess their hemodynamic performance, encompassing key parameters such as head, flow, and hemolytic properties. In this paper, we undertake a systematic review of the evolution of this technology system and propose a three-level classification model based on bibliometric analysis (*n =* 130), in which the dual-circulatory system accounts for 47.27% of the total, to reveal its physiological synergistic mechanism and the innovative application of multi-circulatory configurations in complex clinical scenarios. The study indicates that the prevailing technological impediments pertain to: (i) deviation of 3D-printed vascular mechanical properties (anatomical fidelity loss), (ii) decline in long-term shear force simulation accuracy, and (iii) paucity of module interface compatibility. From an interdisciplinary integration perspective, the present study indicates that adaptive closed-loop hybrid-MCL systems represent a key direction for technological evolution: their architecture, which couples real-time digital twins with physical loops, can dynamically adjust blood flow parameters. When combined with multi-scale simulation optimization, this approach significantly enhances the reliability of long-term shear stress predictions. Furthermore, the integration of personalized digital twins establishes a high-fidelity patient-specific validation platform, thereby providing a theoretical framework for precise evaluation of cardiovascular devices.

## Introduction

The MCL serves as a foundational experimental platform for cardiovascular research, capable of precise reproduction of physiological parameters such as blood pressure (BP), heart rate (HR), vascular compliance (VC), and resistance (PR), among others. This capability stems from the construction of sub-systems dedicated to body circulation, pulmonary circulation, and coronary circulation (Yang and Wang, 2023; Masoud et al., 2023). An analysis of the system architecture reveals that the MCL consists of two modules: the driver unit and the fluidic circuit. The primary functions of the MCL include performance validation of CADs, dynamic simulation of physiological and pathological states, and *in vitro* pre-evaluation of therapeutic regimens. Although heart transplantation remains the gold standard therapy for end-stage heart failure, CAD implantation has become an important alternative due to donor shortages and surgical risks (Huang, 2013; Liang, 2016; Libera et al., 2022; Yang and Wang, 2023). In this context, the MCL provides a controlled and reproducible *in vitro* validation environment for CADs, and is particularly irreplaceable in hemodynamic optimization and device stability testing.

The conceptual prototype of MCL originated with the development of the first artificial heart-lung device by Gibbon in 1935 (Silvay and Castillo, 2013; Flick, 1957; Böttcher and Alexi-Meskishvili, 2006; Pastuszko and EdieGibbon, 2004), and the early systems were based on nonpulsatile flow simulation, focusing on the mechanical reproduction of vascular resistance characteristics (Makinouchi et al., 1994; Yoshino et al., 2001). Typical representatives, such as the primary model constructed by Yoshizawa et al. (2002) used a reservoir vessel to simulate venous return and regulated peripheral resistance through a pinch stop valve. Such systems are only capable of evaluating the basic performance of blood pumps due to the lack of a cardiac pacing module and the limitation of anatomical accuracy, and have been gradually iterated as research needs have increased.

**FIGURE 1:**
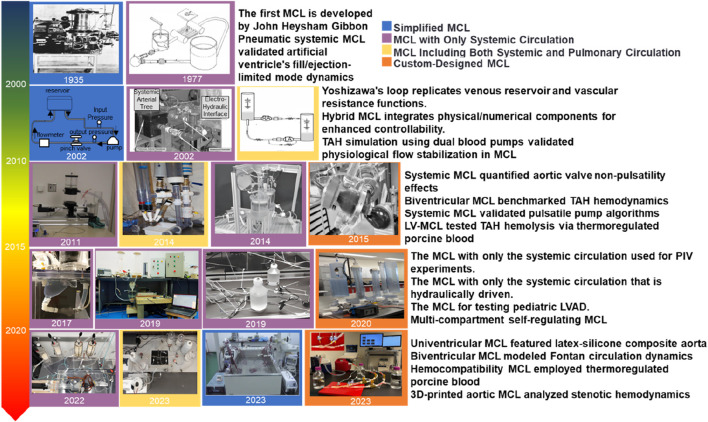
Overview of the different stages of MCL development (Ferrari et al., 2002; Geselowitz et al., 1977; Pastuszko and EdieGibbon, 2004; Yoshizawa et al., 2002; Khalil et al., 2008; Tuzun et al., 2011; Jahren et al., 2014; Love et al., 2014; Gräf et al., 2015; Reider et al., 2017; Pugovkin et al., 2019; Gregory et al., 2020; Alavi et al., 2022; Hong et al., 2023; Li et al., 2023; Rasooli et al., 2023; Khienwad et al., 2019).

A categorization of MCL-related studies, as depicted in Figure 1, is proposed based on the history of MCL development and the literature cited in the article. The categorization system includes four types: simple MCLs, MCLs with body circulation only, MCLs with body circulation and pulmonary circulation, and specially designed MCLs. The latter category has significantly expanded the boundaries of clinical applications of MCLs through the introduction of technological innovations such as 3D printing and smart materials. The developmental timeline of MCLs is illustrated in Figure 1.

The MCL structure is composed primarily of a driver and a circulatory loop. The driver serves as the power source for the heart’s pumping of blood and is frequently employed to simulate the atria and ventricles when integrated with the chambers. The circulatory loop is utilized to simulate an array of components external to the heart, such as blood vessels and valves, within the context of blood circulation. The amalgamation of the drive system and the circulatory loop facilitates a comprehensive simulation of the human body. The integration of these systems enables the full simulation of blood circulation within the human body. Through the modification of the parameters that define the components of the MCL, the simulation of diverse physiological states, including sedentary, exercise, and hemodynamic states associated with cardiovascular diseases such as heart failure and single ventricle, can be achieved. The evolution of the MCL, influenced by the advancement of related fields such as materials science, has led to modifications in its hardware and structural materials. The integration of these components often facilitates the simulation of atrial ventricles. The classification of components has undergone several modifications, with the components now being categorized into three distinct types: traditional, innovative, and other. The development of the MCL’s structure is outlined in Table 1.

**TABLE 1:** Overview of MCL structural development.

MCL structural components	Traditional	Innovative	Other types
Drive systems	Motor drives (Bazan and Ortiz, 2016; Guo, 2022; Vignali et al., 2022) Gas drive (Kado et al., 2021; Malone et al., 2022; Kado et al., 2020; Choi et al., 2014) Hydraulic drive (Mi et al., 2023)		
Vascular	Silicone hose (Jeong et al., 2020; Tozzi et al., 2017)	3D printed blood vessels (Jeong et al., 2023; Guo, 2022; Maryakhina, 2023; Luo, 2016; Chen et al., 2023) Artificial blood vessels (Ferrari et al., 2019; Amabili et al., 2020)	Real animal blood vessels (Reil et al., 2023)
Arterial compliance	Elastic chamber (Gregory et al., 2020; Agrafiotis et al., 2021)		
Vascular resistance	Pinch point valve (Fajdek and Golnik, 2010; Cardiovascular Mathematics, 2010; Li et al., 2020a; Bender et al., 2023; Nestler et al., 2014)		Parallel pipe (Kung and Taylor, 2011; Leopaldi et al., 2012) 3D printed porous materials (Franzetti et al., 2019)
Valves	Check valve (Gregory et al., 2020; Tuzun et al., 2011; Gräf et al., 2015)	Artificial valve (Khienwad et al., 2016; Gehron et al., 2019; Tanné et al., 2010; Bazan and Ortiz, 2016; Marassi et al., 2004)	Real animal valves (Xu, 2017; Kim et al., 2021)
Atrium ventricle	Drivers and chambers (Franzetti et al., 2019)	3D printed or otherwise fabricated hearts (Malone et al., 2022; Liu et al., 2021; Tanné et al., 2010; Brunner et al., 2022)	Real animal heart (Granegger et al., 2013; Schampaert et al., 2013; Baturalp, 2016; Meskin et al., 2024)
Blood	Water and glycerine (Hildebrand et al., 2022; Yilmaz and Sedef, 2023; Shehab et al., 2019; Nestler et al., 2014) Water and other mixtures (Kadem et al., 2005; Marassi et al., 2004; Nguyen And et al., 2004)		Real blood (Gräf et al., 2015; Garrison et al., 1994a; Roka-Moiia et al., 2020)

The prevailing focus of MCL research has undergone a shift towards the latter three types of systems. Early models, which were simple in nature and based on venous fluid storage and simple resistance regulation, have limited research and clinical value. This is due to their inability to effectively simulate complex cardiovascular disease states and comprehensively test modern CADs. The extant review literature has clear limitations. For instance, Agrafiotis et al. (2024). concentrated exclusively on total artificial hearts (TAH) and left ventricular assist devices (LVAD). Moreover, the mechanical/numerical/hybrid three-part rule proposed by Cappon et al. (2021) lacks systematic adaptation analysis for different types of CADs.

In order to address this gap, this study proposes and adopts a “CAD type-oriented” review framework as a novel approach. The objective of this framework is to methodically trace the developmental trajectory of MCL technology and elucidate the fundamental compatibility relationships between diverse MCL systems (e.g., systemic circulation only, systemic-pulmonary circulation, special designs) and mainstream CADs (including ventricular assist devices (VADs), intra-aortic balloon pumps (IABP), TAH, etc.). This compatibility analysis framework has been used to identify three cutting-edge directions that are required to overcome the current technical limitations. Firstly, there is a necessity for adaptive closed-loop hybrid-MCL systems that solve dynamic response bottlenecks through digital-physical real-time coupling. Secondly, multi-scale simulation accuracy optimization is required, which combines computational fluid dynamics and organ-on-a-chip technology to improve the reliability of long-term shear force prediction. Thirdly, there is a requirement for personalized digital twin integration that relies on patient imaging data to build a high-fidelity verification platform. This framework provides a systematic solution for the precise evaluation and clinical translation of cardiovascular devices.

## MCLs for CADs testing

The validation of the performance and stability of VADs—a critical medical device for enhancing cardiac output in patients with heart failure—is contingent on the MCL (Li S. et al., 2020; Rosalia et al., 2021; Xu, 2017; Ochsner et al., 2013; A et al., 2023). A standard MCL comprises left ventricular chamber, aortic valve, mitral valve, and arterial compliance modules, which can be utilized to emulate the physiological and pathological states of the cardiovascular system (CVS). This capability facilitates the assessment of VAD functionality and the optimization of control strategies. *In vitro* MCL testing offers a reproducible validation platform for device performance while ensuring patient safety, in comparison to *in vivo* experimentation, which carries ethical risks and financial costs. A review of the literature indicates the effectiveness of existing MCL studies in evaluating the hydrodynamic properties of CADs under multiple cyclic loads. Despite the discrepancy between MCL-generated pressure waveforms and human data, its *in vitro* test results are valuable in guiding the preclinical validation of CADs. It is important to note that the integration of a hemodynamic monitoring module, additive manufacturing technology, and a multi-sensor fusion system enables the MCL to enhance the ability to dynamically resolve the working state of CADs and optimize the device control algorithm. This chapter will systematically explore the application characteristics of different MCL systems in device performance testing and control strategy development, based on the CADs classification framework.

## MCLs for ventricular assist device testing

**FIGURE 2:**
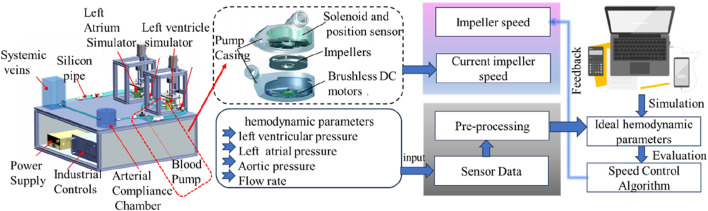
MCL workflow for testing LVADs.

**FIGURE 3:**
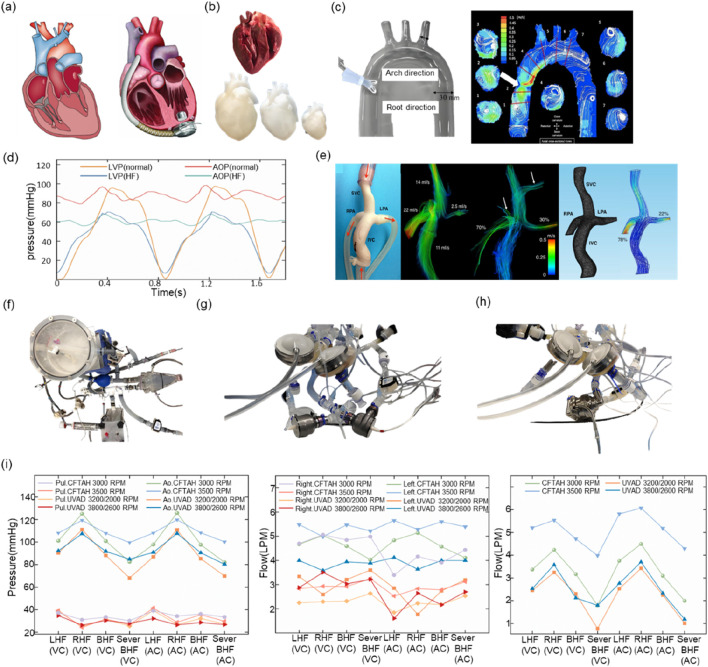
**(a)** Heart failure heart vs. heart implanted with LVAD **(b)** Isolated porcine heart vs. 3D-printed heart **(c)** PIV experiment **(d)** Left ventricular pressure vs. aortic pressure in normal vs. heart failure state **(e)** Fontan circulatory model vs. 4D Flow MRI effects **(f)** MCL used to test IABP **(g)** MCL used to test BiVAD **(h)** MCL used to MCL for testing TAH **(i)** BiVAD vs. TAH at the same MCL.

LVAD is a core intervention for the treatment of left heart failure by driving the cyclic flow of blood from the left ventricle to the aorta via an impeller (Leopaldi et al., 2015), and the internal state of the heart in heart failure with the cross-section of the heart after LVAD implantation is shown in Figure 3a. MCL-based *in vitro* testing of LVADs includes three main directions: hydrodynamic performance evaluation, control algorithm optimization and complication simulation studies. Notably, clinical complications such as thrombosis, ventricular pumping and valvular regurgitation may be induced after LVAD implantation due to the altered hemodynamic environment, which makes the value of MCL in the study of pathomechanisms particularly prominent (Rogers et al., 2017). The content and flow of the MCL workflow for testing LVAD is shown in Figure 2.

In the domain of LVAD performance assessment, Xu (2017) successfully simulated the hemodynamic characteristics of LVAD in dilated heart failure by modifying the Windkessel four-component MCL model while preserving the physiological function of the right heart-pulmonary circulation and optimized the axial-flow pump characteristic curves accordingly. Shu Li’s team (Li S. et al., 2020) proposed the concept of systematic validation, constructed the test matrix that included 12 physiological-pathological conditions, and systematically analyzed the interaction mechanism between LVAD and the circulatory system through the integration of the MCL platform of the body/pulmonary dual circulation. In a related study, Wilson et al. (2023) and Tozzi et al. (2017) employed a glass-blown, transparent left ventricle model in conjunction with particle image velocimetry (PIV) technology to visualize and monitor the end-flow field (Reider et al., 2017; Roldán-Alzate et al., 2015; Goto et al., 2021; Tozzi et al., 2017). Furthermore, certain studies have utilized isolated porcine hearts as an alternative to conventional hydraulic actuators, a method that can more accurately replicate the mechanical characteristics of the heart. However, its implementation and advancement remain constrained due to the intricacy involved in acquiring and maintaining biological specimens (van Dort et al., 2020; van Dort et al., 2022; Granegger et al., 2013; Meskin et al., 2024). Figure 3b presents a comparison between an isolated porcine heart and a 3D-printed heart.

Existing studies have primarily centered on the utilization of the MCL to emulate particular hemodynamic conditions or to examine the mechanisms through which the LVAD interacts with the circulatory system. However, exhaustive investigations of complications subsequent to LVAD implantation frequently necessitate reconfiguration of the MCL hardware or adaptation of control strategies. Thrombosis is closely related to the flow field perturbation inside the LVAD, so most studies have used transparent flow channel design combined with PIV technology for flow field visualization (Goto et al., 2021; Reider et al., 2017; Roldán-Alzate et al., 2015), as shown in Figure 3c for the experimental image of PIV (Roldán-Alzate et al., 2015). In terms of hemolytic effect assessment, researchers have predominantly employed quantitative analysis of blood damage by integrating microscopic imaging and spectrophotometric detection modules (Giridharan et al., 2011; Leme et al., 2011; Woelke et al., 2020). Furthermore, LVAD is susceptible to inducing ventricular pumping or pulmonary circulation stasis due to its inherent characteristics of sluggish preload response and enhanced afterload sensitivity. To mitigate these risks, closed-loop control algorithms are necessary (Ochsner et al., 2013; Gregory et al., 2020; Rocchi et al., 2023a). Tuzun et al. (2011) revealed the dynamic effects of LVAD operating parameters on aortic valve function based on the MCL platform, and the results of Y. Tanaka’s team (Tanaka et al., 2020) further used a silicone LVAD model to validate the effectiveness of the pump speed-blood pressure synergistic optimization strategy for the intervention of severe aortic regurgitation.

It is noteworthy that epidemiological data on heart failure in children indicates that approximately 12,000–35,000 children require cardiac support each year in the United States. Their high readmission rates and treatment costs underscore the urgency of pediatric LVAD development. Given the anatomical structure and hemodynamic characteristics of the cardiovascular system in children, which are significantly different from those of adults, the development of a dedicated pediatric MCL system is essential to meet the needs of device testing. Gregory et al. (2020), Vandenberghe et al. (2011), and Huang et al. (2013) the authors successfully achieved the *in vitro* functional validation of pediatric ventricular assist devices by constructing scaled-down MCLs. These MCLs have the advantages of channel sizing and low-flow simulation accuracy, and have the potential to provide a more accurate and efficient device. Tuning and low-flow simulation accuracy with unique technical advantages.

The classification of VADs is determined by the therapeutic objective, with LVADs, right ventricular assist devices (RVADs), biventricular assist devices (BiVADs), and interventional ventricular assist devices (PVADs) being the primary categories. The evaluation of RVADs and BiVADs necessitates the simulation of both the physical and pulmonary circulations, due to the heterogeneity of the disease conditions targeted. Right heart failure is frequently a secondary condition resulting from LV dysfunction, and its pathogenesis involves multiple factors, including altered venous return, septal dyskinesia, and elevated pulmonary artery pressures due to LVAD. As an intervention for right heart failure, the Fontan procedure can achieve direct vena cava-pulmonary artery anastomosis through the creation of a Total Cavopulmonary Connection (TCPC). While this approach enables the direct vena cava-pulmonary artery anastomosis, it also introduces the vulnerability of Fontan circulation failure due to the absence of right heart pump function. To address this limitation, the clinical implementation of an RVAD has emerged as a therapeutic strategy to manage or alleviate Fontan circulatory failure. Roldán-Alzate et al. (2015) employed 4D flow magnetic resonance to compare the flow field characteristics of real blood vessels with those of a 3D-printed model, thereby providing a validation basis for the numerical simulation of TCPC. A representative 4D Flow MRI image of the TCPC configuration is shown in Figure 3e. Gregory et al. (2020) demonstrated by the RVAD-MCL system that the effectiveness of the device to reduce inferior vena cava pressure, but the study failed to fully evaluate the effect of blood composition on device performance due to the rheological differences between experimental fluids and real blood.

In the case of biventricular failure, the available clinical options for mechanical support include the BiVAD and the TAH. In order to enhance the fidelity of blood utilized in testing (Kado et al., 2020), the ANASTASIOS PETROU team (Petrou et al., 2019) employed a bovine blood-human blood mixture as the circulating medium, in conjunction with a hydraulic/pneumatic composite drive system, to effectively simulate the effects of biventricular assist in complex pathologies such as aortic regurgitation. Notably, Nguyen And et al. (2004) have pioneered the integration of the SynCardia TAH single-pump chamber into the Fontan circulating MCL, thereby demonstrating the feasibility of the device to substitute for right heart function and offering novel insights into single ventricle assist strategies.

PVADs have been shown to offer distinct advantages in the context of short-term circulatory support, typically ranging from 0 to 15 days, due to their unique characteristics related to percutaneous implantation, which is typically accomplished via the femoral or brachial artery route. To address the challenges posed by the complex flow field characteristics within the PVAD, Gupta et al. (2020) have developed a body-circulating MCL with an integrated PIV module, in conjunction with a 3D-printed pump casing, for the purpose of visual monitoring. This module enables the visualization of the flow field. Monreal’s team established a PVAD testing platform that is clinically verifiable by adjusting the system resistance through Hoffman clips in conjunction with a constant-temperature blood-simulating fluid (Monreal et al., 2023). *In vivo* experimental data were in good agreement with the MCL test results. These studies provide an important *in vitro* validation tool for optimizing the performance of VADs with different interventional pathways.

The application of MCLs in different types of VADs demonstrates that traditional MCLs have been more developed, and today’s MCLs can be flexibly assembled or replaced with powerful functional modules, such as PIV, artificial heart, and VADs modules, according to different needs. However, there is a paucity of studies related to MCLs developed for children. Nevertheless, it is hypothesized that this phenomenon will promote the development of related MCLs in the future, as the number of children with cardiovascular diseases is increasing year by year. A review of the literature reveals that specially designed MCLs will be more emphasized in future clinical applications to accomplish more complex requirements.

## MCL for intra-aortic balloon counterpulsation testing

IABP is a CAD that is commonly used to assist cardiac recovery, improve coronary circulation, and reestablish the balance of oxygen supply and demand (Ferrari et al., 2001; Ferrari et al., 2011; Ferrari et al., 2005). It does so by improving coronary perfusion and regulating the balance of myocardial oxygen supply and demand through time-phased balloon contraction and relaxation. The intervention is typically performed by positioning a balloon catheter via the femoral or external iliac artery in the descending aortic segment between the distal 1–2 cm of the left subclavian artery and the proximal renal artery. Hemodynamic regulation of diastolic pressurization and systolic decompression is achieved by an extracorporeal counterpulsation controller (Patterson et al., 2014; Khan and Siddiqui, 2022; Xu et al., 2023). Wave intensity analysis, a pivotal parameter for assessing the efficacy of IABP, can differentiate between forward propagating waves and reverse reflected waves by employing time-domain features, thereby providing a quantitative basis for device optimization (De Lazzari et al., 2023; Capoccia et al., 2015; Kolyva et al., 2009; Lu et al., 2011; Parker and Jones, 1990; Bleasdale et al., 2003).

Since the introduction of IABP into the clinic by Capoccia et al. (2015) and Kantrowitz et al. (1993) in the 1960s, it is *in vitro* testing technology has continued to evolve. The first MCL, established in 1971, had basic the evolution of IABP-MCL testing capabilities has been driven by the demand for developing devices such as LVADs. This demand has led to the development of modern IABP-specific MCLs, which have gradually incorporated coronary artery circulation simulation, silicone 3D-printed aortic models, and other bionic components. In contrast to other test platforms for ventricular assist devices, IABP-MCL requires the integration of an artificial aortic module and a synchronized drive (Xu et al., 2023). While earlier systems only simulated vascular compliance and resistance parameters through elastic lumens and pipelines, current state-of-the-art models have enabled multiscale simulation from the atrial ventricle to the systemic-coronary circulation, which significantly improves the biofidelity of hemodynamic simulation. Similar to other VADs, IABP-MCL requires a closed-loop control system to precisely regulate the timing of balloon inflation and deflation to ensure optimal mechanical coupling with the cardiac cycle.

Mechanical implantable devices, including LVAD, IABP, and IARBP, have been utilized as adjunctive therapy for patients with heart failure. Beyond the fundamental components of the MCL, including the drive system, the atria, and the valves, Wang et al. (2017) and Cappon et al. (2021) have explored the potential of concurrently testing multiple CAD models. This approach was undertaken to enhance the comparability of the data by developing an MCL that incorporated the brachiocephalic trunk, the subclavian artery, the common carotid artery, and the coronary arteries. Building upon this approach, Ferrari et al. (2001) refined the MCL, incorporating it into LVAD and BiVAD testing to enhance its compatibility with IABP evaluation. In their model, the aorta of the MCL was replicated by a conical rubber tube. Due to the complexity of the human body’s positioning, it is often necessary to evaluate the performance of the IABP in various states after implantation procedures. To address this issue, Swalen et al. (2005) studied the performance of the IABP in the tilted state, the IABP was placed in a real-sized latex aorta in an MCL operated horizontally at an angle of 10°, by tilting the artificial aorta to achieve this. More recently, Kolyva et al. (2016) and Kolyva et al. (2012) designed an MCL that simulated and tested IABP performance in multiple patient positions by modeling the aorta with a 12-branch polyurethane composite aorta and with all branches linked at the capillary and other vascular structures, The end resistance and end compliance of the model were provided by the aforementioned branches, respectively. In contrast, Corazza et al. (2014) and Corazza et al. (2013) departed from the traditional idea of the MCL for testing IABP by innovatively incorporating an external reservoir to amplify balloon pulsations induced by physiologic arterial pressure pulses.

In the context of evaluating mechanical implantable assist devices, it is imperative to acknowledge the inherent differences in hemodynamic parameters, such as aortic pressure and flow, between children and adults. Consequently, MCLs must be deliberated upon in consideration of diverse application populations. Pantalos et al. (2010) introduced a 5 cc pediatric IABP catheter through an O-ring sealed connector in their designed MCL and into the thoracic descending aorta through an iliac bifurcation Y connector to simulate the introduction and location of IABP in the aorta of pediatric patients (Wang et al., 2017; Cappon et al., 2021; Pantalos et al., 2010).

By examining the evolution of the MCL utilized for IABP testing from its early iterations to the contemporary MCL, it becomes evident that the MCL employed for IABP testing has evolved in a manner analogous to the broader development of the MCL with respect to its structural composition, and the hardware can be upgraded according to the updating of the materials of the aorta and other components of the MCL. At the practical application level, the performance of the IABP is limited in two aspects due to its structural characteristics. Firstly, the diameter of the balloon catheter must be sufficiently small due to surgical constraints. Secondly, the compressibility of the gas, from which the IABP is less efficient, is another factor (De Lazzari et al., 2023; Capoccia et al., 2015). Consequently, in the actual treatment process The IABP is commonly used as a short-term means of support to create the conditions for the ventricle to recover benign circulation, thereby eliminating the need for long-term support (Xu et al., 2023).

## MCL for testing total artificial hearts

TAH is distinguished from VADs in that it comprises four components: the VADs, driver, monitoring system, and energy source. The TAH has the capacity to substitute for the right and left ventricles following implantation in the human body, thereby artificially facilitating both body and pulmonary circulation. Consequently, the MCL must encompass both body circulation and pulmonary circulation (Kuroda et al., 2023). In terms of application targets, the total artificial heart and BiVAD have similarities and are more suitable for application in patients with right ventricular failure caused by LVAD implantation. In order to understand the facilitating effect of the total artificial heart and BiVAD on patients with heart failure, respectively, Karimov et al. (2023) compared the hemodynamic effects of the total artificial heart and the BiVAD by establishing two different types of MCLs. The hemodynamic performance of the BiVAD is illustrated in Figures 3g,h.

The initial MCL utilized for the study of TAH was developed by Kolff et al. (1959) this initial model lacked the capacity to regulate resistance, thereby constraining the scope of TAH testing (Kolff et al., 1959). Subsequently, Laumen et al. (2013) employed electrically adjustable components in the MCL. This MCL was designed to assess the hemodynamic performance of TAH to simulate physiological, pathological, and particularly changing circulatory conditions. A bronchial shunt was incorporated into the MCL bypass, and the overall performance of the TAH was evaluated *in vitro* with this configuration. Notably, no thrombus was generated during the prolonged testing period. To achieve an optimal left-right blood flow balance, Ng et al. (2017) developed an adaptive starlink-like controller based on a four-element MCL. This controller incorporated an adaptive mechanism to minimize the risk of pulmonary congestion and atrial pumping, while meeting the cardiac pumping demands. In contrast to the MCL design by Vignali et al. (2022) the proposed controller utilizes a technical atrial shunt (TIAS) to enable independent testing of the left and right outputs of the TAH. This TIAS compensates for volume shifts between the pulmonary and somatic circulations due to potential blood flow imbalances.

As with MCLs for testing VADs, MCLs for testing TAHs can similarly be assembled and replaced with different modules to simulate more human states. For instance, as reported by Love et al. (2014) the circulatory loop was designed to include a number of novel features, including pressure-regulated slots for These include the incorporation of pressure-regulated slots to simulate exercise conditions, the inclusion of adjustable hardware parameters, and the incorporation of pulmonary vascular resistance to simulate changes in human exercise status and left-right flow imbalance during respiration. Additionally, a left atrial suction valve has been incorporated. Garrison et al. (1994b) reported that left ventricular blood from TAH has a higher hemolysis rate than ventricular blood from the pulmonary circulation. Consequently, Gräf et al. (2015) investigated hemolysis in the left ventricle of TAH by designing an MCL for the observation of blood compatibility. In this model, porcine blood was used as the fluid, and the atria were connected to the ventricles to This approach was undertaken to minimize the effects of fluid inertia. In contrast to the majority of MCLs, which do not incorporate a non-invasive pressure chamber into their structural design, this study’s MCL was equipped with such a chamber, ensuring the measurement of flow or pressure without direct blood contact. The interior of the MCL was constructed with blood-compatible materials, thereby preventing direct contact between the blood and the air. The study culminated in a final test of blood compatibility with TAH support, which was performed to assess the effectiveness of the designed MCL.

According to the literature review, the key aspects of testing TAH include adequate cardiac output, balanced left and right flows, performance testing, and blood compatibility. However, there are currently fewer studies related to blood compatibility for TAH. TAH for the other testing aspects. It is hypothesized that in the future, there will be more MCL experiments on blood compatibility for TAH due to the increasing emphasis on the blood-destructive nature of the mechanical implants. Blood compatibility will become a major concern.

## Hybrid-MCL for personalized CAD assessment

In addition to the aforementioned fully physical MCLs, Hybrid-MCLs have emerged in recent years, revolutionizing testing of cardiovascular devices by combining real-time computational models with physical fluid circuits (Bardi et al., 2022). This architecture dynamically couples lumped-parameter digital twins to hydraulic interfaces via high-bandwidth sensors, extending beyond personalized hemodynamic tuning to encompass dynamic pathological event simulation—particularly critical arrhythmias affecting over 50% of VAD patients (Hong et al., 2025; Cappon et al., 2021; Korn et al., 2018).

Fresiello’s foundational work demonstrated patient-specific tuning across clinical phenotypes with <10% error (Fresiello et al., 2015), while recent advances by Rapp et al. (2022) have addressed the critical gap in transient pathology modeling through ECG-driven approaches. By processing fiducial points from clinical ECGs (PhysioNet MGH/MF database), their system modulates ventricular elastance functions to replicate atrial/ventricular fibrillation, achieving <4% pressure tracking error during VAD-supported arrhythmias. This multi-method framework combines: real-time CVS parameter adjustment for acute stenosis induction; clinical PLV data tracing for atrial fibrillation; and F-wave injection (8 Hz mean frequency) for electrophysiological fidelity.

Building on these personalized and pathological simulation capabilities, Ochsner et al. (2012) engineered a standardized numerical-hydraulic interface operating at 5 kHz. This enables real-time assessment of mixed-flow VADs under pulmonary hypertension (PH) and exercise conditions without physical reconfiguration. Concurrently, Bardi et al. (2024) expanded this paradigm to anatomical fidelity: their PID-controlled Windkessel outlet generates patient-specific waveforms, while LED-PIV technology captures 3D velocity fields in a compliant aneurysm model, thereby demonstrating that hybrid-MCL can simultaneously resolve global hemodynamics and local fluid dynamics. In the context of complex biventricular applications, Perra et al. (2025) implemented a preload-responsive controller in a TAH-guided hybrid-MCL. This controller autonomously adjusts impulse parameters during sleep-rest-exercise transitions while maintaining flow balance during pulmonary artery pressure fluctuations. In a recent study, Nair et al. (2025) validated the applicability to congenital diseases. The pressure drops in a compliant aortic stenosis model matched 3D fluid-structure simulation results with an average error of only 1.6 mmHg, demonstrating robustness in pediatric motion states.

It is evident that these advancements establish hybrid-MCL as a unified platform that integrates computational adaptability and physical realism. This ultimately accelerates the optimization of patient-specific devices through repeatable *in vitro* tuning and clinical validation.

## Discussion

The MCL has become the primary human hemodynamic simulation system for evaluating cardiovascular implantable devices, including ventricular assist devices and total artificial hearts. The simulation of hemodynamic changes following device implantation, as conducted by MCL, is a critical step in validating device performance prior to surgical intervention. It is evident that the implementation of continuous improvements to the MCL’s structural framework has resulted in a notable expansion of its application scope, thereby encompassing complex clinical scenarios with increasing complexity. The integration of modular designs and Hybrid-MCLs has emerged as a pivotal technological catalyst for this progress. For instance, by replacing or expanding modules (e.g., the aorta, ventricular assist device, or total artificial heart), MCL can adapt to the testing requirements of different devices. Furthermore, hybrid systems facilitate dynamic simulation of pathological states by real-time coupling of digital cardiovascular models with physical circuits. It is important to note that total artificial hearts require replacement of both left and right ventricular structures, while ventricular assist devices retain partial cardiac function. This discrepancy leads to substantial variations in the extraction of circulatory parameters across different devices. The digital twin architecture of hybrid-MCLs provides a unified solution by adjusting digital model parameters, thereby enabling the rapid switching of device testing scenarios and circumventing the hardware reconfiguration bottleneck inherent in traditional MCLs (Malone et al., 2022).

Since the advent of MCL, an analysis of prevailing trends has revealed that the complexity of MCL manifests a distinct hierarchical structure. In the analysis of the 130 studies included, it was found that approximately 16.36% of MCL models incorporated only the systemic circulation, rendering them suitable for basic parameter testing of left ventricular assist devices. In addition, 47.27% of MCL models integrated both the systemic and pulmonary circulations, thereby enabling simulation of the coupled effects of ventricular assist devices on the cardiopulmonary system. The remaining 36.36% of MCL models further incorporated subsystems such as the coronary circulation, cerebral blood supply, and renal circulation. It is noteworthy that recently emerging hybrid-MCLs integrate multi-circulatory physiological interactions (e.g., cerebral blood flow autoregulation coupled with systemic circulation) through digital modelling, thereby significantly reducing hardware complexity. Despite the prevailing focus on dual-circulatory MCLs in current research, there has been an annual increase in the proportion of multi-circulatory MCLs and hybrid-MCLs. This trend may be closely related to clinical concerns about the long-term safety of devices, such as the potential for blood cell damage caused by impeller rotation in ventricular assist devices, as well as complex cases, such as the customized needs of amputees for extracorporeal simulation. The primary benefit of hybrid systems is the ability to achieve “virtual multi-circuit coupling” through the utilization of digital twin technology. This approach circumvents the occurrence of physical interface compatibility issues while concurrently supporting personalized hemodynamic validation for complex cases, such as those involving patients with arrhythmia. Furthermore, although the number of publications in 2025 has slightly decreased, the overall annual publication trend continues to show a linear increase, indicating sustained growth in research activity in this field.

However, current MCL research still faces numerous technical challenges. The first is the compatibility issue of modular design. The fluid dynamics parameters of different circulatory loops must be dynamically matched. For example, when replacing circulatory modules for organs such as the brain or kidneys, pressure adjustments are necessary to prevent system imbalance. The brain circulatory system is just one typical scenario; similar challenges exist in subsystems such as the liver and kidneys. Additionally, the interface sealing and anticoagulant properties of 3D-printed organ models and MCL hardware require further optimization (Xu et al., 2023). Notably, the FDA-led Round Robin study (aimed at harmonizing testing protocols for mechanical circulatory support devices) will provide a critical framework for addressing multi-center data comparability, long-term performance evaluation, and module interface standardization, and is expected to guide revisions to the next-generation of the ISO 14708-5 standard (Fresiello et al., 2015; Fresiello et al., 2024; Rocchi et al., 2023b; Fresiello et al., 2014). Hybrid-MCL offers an innovative approach—digital models assume parameter coordination functions (e.g., Fresiello’s closed-loop regulation), while physical circuits only need to maintain basic interfaces, significantly reducing hardware adaptation complexity. Second is the limitation of long-term performance evaluation. Existing MCLs are primarily used for short-term testing, but ventricular assist devices must operate continuously for years after implantation. How to simulate long-term wear through accelerated aging experiments remains a challenge. Hybrid systems can simulate pathological evolution processes spanning months or even years by integrating digital degradation models (e.g., Perra’s physiological controller). Finally, there is the complexity of multi-loop coupling. For example, the introduction of brain blood flow autoregulation mechanisms may disrupt system stability. Next-generation hybrid-MCLs use intelligent algorithms (such as Rapp’s ECG-driven elastic function (Rapp et al., 2022)) to dynamically adjust digital-physical interactions in real time, significantly enhancing system robustness.

In the future, hybrid-MCL will become the key paradigm for overcoming technical limitations. The digital architecture of the system is of a modular nature, thus supporting the concept of “virtual plug-and-play.” To provide an example, in the case of amputees, it is only necessary to adjust the digital circulation parameters, whilst the simulation of intracranial vascular lesions can be achieved through the hybrid integration of 3D-printed models and PID controllers. Of greater significance is the enhancement of MCL, which transitions from a hardware testing instrument to a clinical decision-making platform. This transformation is exemplified by the integration of patient imaging data to generate digital twins and the simulation of the hemodynamic response subsequent to device implantation (for instance, the efficacy of aortic valve stenosis intervention). Furthermore, cross-scale simulation (for example, computational fluid dynamics integrated with hybrid-MCL) and machine learning optimization will propel personalized validation into a new phase. Despite the fact that research on these fusion technologies is still in its infancy, their value in reducing clinical risks and accelerating device translation is becoming increasingly apparent.

## Conclusion

The evolution of MCL has been predominantly driven by the demands of CADs, with a shift from single-function validation to multi-organ physiological simulation. A review of the literature from the past decade reveals that MCLs encompassing body circulation and pulmonary circulation remain the prevailing standard. However, there has been an observed annual increase in the prevalence of multi-circulation MCLs. This trend mirrors the clinical concern regarding the long-term safety of CADs and the impact of multi-organ interactions. The integration of 3D-printed organ models with multimodal sensors, such as PIV and 4D Flow MRI, is facilitated by modular design in MCLs. However, significant technical challenges persist. Insufficient fluid compatibility of multi-loop circuits (i.e., dynamic matching capability of pressure-flow parameters between subsystems), such as the conflict between cerebral circulation autoregulation and other circulatory systems, remains a substantial hurdle. Simulation and mechanical pumping control; second, the existing MCLs are difficult to simulate the biocompatibility problems caused by long-term blood flow shear, such as the VAD impeller’s blood cell damage; and third, low matching of individualized physiological characteristics, which restricts the efficiency of clinical translation. In response to these challenges, research indicates that adaptive closed-loop hybrid-MCL systems represent a critical breakthrough pathway. These systems dynamically coordinate multi-loop conflicts through real-time digital twin coupling with physical loops. Simultaneously, multi-scale simulation accuracy optimization significantly enhances the reliability of long-term shear force predictions. This is achieved through a computational fluid dynamics and organ-on-a-chip integrated platform. The integration of personalized digital twins establishes a high-fidelity validation environment by integrating patient imaging and postoperative data, thereby systematically addressing issues pertaining to physiological feature matching. The overarching objective of MCL has exceeded the confines of conventional *in vitro* validation frameworks, metamorphosing into a physiological digital twin that serves as a nexus between engineering design and clinical decision-making. This transformation necessitates interdisciplinary collaboration to drive technological innovation and standardized integration.
